# Effects of Acorns on Fatty Acid Composition and Lipid Metabolism in Adipose Tissue of Yuxi Black Pigs

**DOI:** 10.3390/ani14223271

**Published:** 2024-11-13

**Authors:** Zhe Sun, Dongyang Liu, Siyuan An, Xuejia Wu, Jinzhou Zhang, Zhiguo Miao

**Affiliations:** College of Animal Science and Veterinary Medicine, Henan Institute of Science and Technology, Xinxiang 453003, China; sz18437911393@163.com (Z.S.); liudy97@126.com (D.L.); asy15903076062@163.com (S.A.); 15038499973@163.com (X.W.); zhangjz69@126.com (J.Z.)

**Keywords:** acorns, Yuxi black pigs, lipid metabolism, FAs

## Abstract

The objective of the present research was the examination of how varying proportions of an acorn diet affects the deposition of subcutaneous fat and the composition of fatty acids (FAs) in Yuxi black pigs. Ninety pigs (with a balanced sex ratio and a similar weight 99.60 ± 2.32 kg) were stochastically assigned to the control group (CON) and the dietary acorn experimental groups (AEG). The CON was fed basal diets and the AEG1, AEG2, AEG3, and AEG4 groups were provided with dietary regimens comprising twenty, thirty, forty, and fifty per cent acorns, respectively. Each group consisted of six pigs, with three replicates. The breeding cycle was four months. The results demonstrated that, in comparison with the CON group, the lean meat rate was significantly increased in all test groups (*p* < 0.05), while in backfat thickness, loin eye area, carcass weight and slaughter rate was no significant difference (*p* > 0.05). The serum TC/HDL (total cholesterol divided by high-density lipoprotein-cholesterol) and TG/HDL (Triglyceride divided by high-density lipoprotein-cholesterol) levels in the AEG1 and AEG2 groups were significantly lower than the CON group (*p* < 0.05). There was no significant effect on the composition of FAs (*p* > 0.05). The number of fat cells in subcutaneous back fat and subcutaneous abdominal fat was significantly increased, and the area of fat cells was decreased (*p* < 0.05). Furthermore, the levels of ATGL and HSL expression in the subcutaneous back fat, as well as ACC, FAS, ATGL, PPARγ, and HSL expression in the subcutaneous abdominal fat, were significantly increased in the AEG2 group compared to the CON group (*p* < 0.05). Additionally, the expression of ACC, FAS, FABP4, PPARγ, C/EBPα, and FAS/HSL in the subcutaneous back fat, as well as FABP4, C/EBPα, and FAS/HSL in the subcutaneous abdominal fat, were significantly lower in the AEG2 group compared to the CON group (*p* < 0.05). In conclusion, it has been found that a 30% acorn diet can inhibit subcutaneous fat deposition and enhance the nutritional value of pork and the health of Yuxi black pigs.

## Simple Summary

With the improvement of living standards, people’s requirements for meat quality are also growing. Reducing backfat thickness and inhibiting subcutaneous fat deposition is crucial for improving the quality of meat. Acorns are rich in nutrients and have antioxidant and hypoglycemic effects. The present study evaluated the role of acorns as a novel feed resource for pig production and found that an acorn diet had positive effects on lean meat rate, blood lipids and the inhibition of subcutaneous fat.

## 1. Introduction

Fat is a major site for storing energy and participating in fatty acid synthesis [1], mainly composing of subcutaneous fat, intramuscular fat, and visceral fat [2]. The distribution and deposition capacity of fat are key factors affecting pork quality [3]. The process of porcine fat deposition showed that the number of fat cells increased in early development and the volume of adipocytes increased in late growth [4]. Fat deposition in pigs is influenced by various factors, such as genetics, nutrition, breed, and age [5]. A previous study showed that excessive subcutaneous fat deposition increases cholesterol levels and reduces pork quality, while an increase in intramuscular fat can improve pork quality [6]. Therefore, the quality and nutritional value of pork can be improved by properly reducing subcutaneous fat and increasing intramuscular fat content.

In China, there are approximately 300 varieties of acorns, most of which are wild and have a wide distribution. They usually mature and fall off in late August to early September. Acorns contain high levels of starch, crude fat, and PUFAs (polyunsaturated fatty acids) [7]. Their nutritional value and calorie content are comparable to that of sorghum and corn. Acorns have antidiarrheal, hypoglycemic, antioxidant effects, and the by-product acorn shells are rich in active ingredients such as polyphenols and tannins, and have been used in food additives [8]. A previous study has found that by increasing the feed intake of acorns, the intramuscular fat (IMF) content of the abdominal muscles of Iberian pigs is increased [9]. In another study, free-range pigs fed silage and a few acorns increased the content of MUFAs (monounsaturated fatty acids) and decreased the thrombogenic index and peroxidability indices [10]. The above research indicates that acorns can serve as a potential new feed resource for pigs.

Yuxi black pigs were formed by natural selection and breeding over a prolonged period of time, and adapted to the local natural and ecological environment. They are predominantly located in the mountainous areas of western Henan, China. On 10 April 2021, the National Livestock and Poultry Genetic Resources Committee of China approved them as a newly discovered and important local livestock and poultry breed in China. Yuxi black pigs are usually raised in captivity and free-range. Compared with modern breeds used in large-scale pig farming, Yuxi black pigs are distinguished by their early maturation, robust immune system, rough-feeding and superior pork quality [11]. The muscle tissue of these pigs contains an abundant amount of fatty acids, with a good ratio of UFAs (unsaturated fatty acids) to SFAs (saturated fatty acids) [9]. However, there are no reports on the effect of dietary acorns on the subcutaneous fat of Yuxi black pigs and its molecular regulatory mechanism. So, can adding acorns to the feed reduce the subcutaneous fat content and regulate pork quality? This study aimed to investigate the effect of acorns on subcutaneous fat content, the composition of FAs, and gene expression related to lipid metabolism in adipose tissue. It provides the foundation for the advancement and implementation of acorn utilization, encompassing their production and application in pig husbandry.

## 2. Materials and Methods

### 2.1. Acorn Preparation

Acorns were purchased from Luanchuan County Heiyuan High Mountain Agriculture and Animal Husbandry Development Company Limited (Luanchuan County, China). After drying and crushing, acorns were added to feed in a certain proportion then mixed and stirred. The nutrition content of acorns is shown in Table 1.

### 2.2. Experimental Design and Diets

Approval for all protocols involving the treatment of test pigs was provided by the Animal Care and Use Committee of the Henan Institute of Science and Technology. (Xinxiang, China). Ninety Yuxi black pigs, with a balanced sex ratio and similar weight (99.60 ± 2.32 kg), were stochastically assigned to the control group (CON) and dietary acorn test groups (AEG). The CON was fed basal diets, and AEG1, AEG2, AEG3, and AEG4 groups were provided with dietary regimens comprising twenty, thirty, forty and fifty per cent acorns, respectively. Each group consisted of six pigs, with three replicates. The breeding cycle was four months. The pigs in each test group were kept in pens of 20 square meters at a temperature of 24 °C and naturally ventilated from June to October. The dietary regimens employed in this investigation were devised in accordance with the guidelines set forth by the National Research Council (NRC, 2012) for the fattening of swine. Table 2 shows the feed composition of each group in this experiment. Table 3 shows the FA composition of each group in this experiment. The pigs that were the subjects of the experiment were provided with sustenance at two distinct intervals: 6:00 and 18:00. The pigs were watered ad libitum during the period and consumed an average of 2.5 kg of the test diets per day.

Premix ingredients per kg: VA: 120,000.00 IU; VD3: 45,000.00 IU; VE: 700.00 IU; VK3: 45.00 mg; VB2: 150.00 mg; VB6: 50.00 mg; niacin amide: 750.00 mg; calcium pantothenate: 460.00 mg; choline chloride: 3.50 mg; copper: 0.31 g; iron: 3.50 g; zinc: 1.42 g; manganese: 0.83 g; iodine: 42.00 mg; selenium: 7.82 mg; ca: 16.00%; phosphorus: 3.53%; lys: 0.2%; cys: 0.05%; met: 0.1%.

### 2.3. Slaughter and Sample Collection

When fed for four months, the pigs are fasted for 24 h and then weighed. Six pigs were selected for each group, two for each replicate for a total of thirty pigs were selected for anterior vena cava blood collection by pro-coagulation tubes, centrifuged at 3500 rpm for 10 min, and plasma was separated and frozen at −80 °C for testing. The slaughter procedure was carried out according to the methods outlined in our previous research [12]. After the blood collection, the pigs were electrocuted, bled, slaughtered, and the indicators related to slaughter performance were measured. Adipose tissue (subcutaneous back and abdomen fat) with a volume of 1 cm × 1 cm × 0.5 cm was taken and fixed with a 10% buffered formaldehyde solution for histological observation. Other samples of fat tissue were placed into 1.5 mL EP tubes and quickly stored in liquid nitrogen for the detection of gene expression related to fatty acid and lipid metabolism. When all samples were collected, they were moved to a −80 °C ultra-low temperature refrigerator.

### 2.4. Slaughter Performance

The average backfat thickness was measured at the thickest point of the shoulder, at the three points of the final rib, and at the waist sacrum junction with a vernier caliper. The loin eye area was calculated by vertically severing the longissimus dorsi at the last rib of the left carcass, placing sulfuric acid paper on the cross-section and drawing an outline along the edge of the oculomotor muscle with a marker pen, and calculating the area with a planimeter. The carcass weight was measured by the weight of the two sides of the body after the head, hooves, tail, and internal organs were removed and the suet and kidneys were retained. The dressing percentage was calculated by the ratio of carcass weight to live weight before slaughter. The lean meat rate was calculated by dividing the carcass lean weight by the lean meat, fat, skin, and bone weight.

### 2.5. Blood Lipid Assay

Triglyceride (TG), total cholesterol (TC), high density lipoprotein cholesterol (HDL-C), and low-density lipoprotein cholesterol (LDL-C) were determined by GOD-PAP method with Hitachi 7020 automatic biochemical analyzer (Hitachi, Tokyo, Japan).

### 2.6. Histometric Analyses

Adipose tissue from six pigs in each group was selected; the adipose tissue of Yuxi black pigs was fixed with a 10% buffered formaldehyde solution; the specimens were dehydrated in graded alcohol and embedded in paraffin at 60 °C inside labelled embedding molds or cassettes. The embedded tissues were sliced on a microtome with a slice thickness of 5 μm, and the slices were floated in a 45 °C water bath to diffuse the wrinkled portion, and then floated on glass slides to allow proper adhesion to the glass slides. Hematoxylin–eosin was then used for staining. Histological examination was conducted using Olympus BX 63 light microscope (Olympus Corporation, Tokyo, Japan), followed by morphological analysis carried out using Image-Pro Plus 6.0 software. At least 20 adipocytes were randomly selected and the diameter and area of the adipocytes were measured in the collected images.

### 2.7. FA Composition Analysis and Indicators of Fat Nutritional Quality

The fatty acid composition was determined using previously reported methods for synthesizing fatty acid methyl esters (FAME) [13,14]. The FAME was analyzed by Gas chromatography (Agilent-7890, Agilent Technologies Inc., Santa Clara, CA, USA) equipped with a flame ionization detector (FID) and a HP-88 capillary column (0.2 µm × 0.25 mm × 100 m, Agilent) [15]. The amount of each fatty acid is calculated as a percentage of peak area of total fatty acids. According to the composition data of FAs, index of atherogenicity (IA), index of thrombogenicity (IT), unsaturation index (UI), hypocholesterolemic/Hypercholesterolemic ratio (h/H), health-promoting index (HPI), and nutrition value index (NVI) of subcutaneous back fat and subcutaneous abdominal fat were calculated using the formulae of previous studies [10,16].

### 2.8. qRT-PCR Analysis

The total RNA was extracted from the tissues utilizing the TRIzol reagent (Invitrogen, Paisley, UK). RNA concentrations and purity were determined by spectrophotometry (IMPLEN, Westlake Village, CA, USA). Complementary DNA (cDNA) was synthesized from approximately 1 μg of the total RNA in each sample using the PrimeScriptTM RT Reagent Kit (Takara Bio Inc., Tokyo, Japan). QuantiFast^®^ SYBR^®^ Green PCR Kit (Qiagen, Dusseldorf, Germany) and ViiA^TM^ 7 real-time PCR system were used to carry out RT-PCR. Table 4 shows the primers required for this experiment. GAPDH was used as the internal reference, and mRNA expression was calculated using the 2^−ΔΔCt^ method.

### 2.9. Statistical Analysis

This study’s data were analyzed using SPSS26.0 software, with one-way ANOVA (analysis of variance) and Duncan’s multiple range test. Data visualization was performed using GraphPad Prism 6 software (GraphPad, San Diego, CA, USA). *p* < 0.05 was considered statistically significant, and the mean ± SEM (Standard Error of the Mean) indicated the result.

## 3. Results

### 3.1. Slaughter Performance

According to Table 5, compared with the CON and AEG1 groups, the lean meat rate of the AEG2, AEG3, and AEG4 groups was increased remarkably (*p* < 0.05). The acorn diet had no significant effects on backfat thickness, loin eye area, carcass weight, and slaughter rate of Yuxi black pigs (*p* > 0.05).

### 3.2. Blood Lipid Index

As illustrated in Table 6, compared with the CON group, HDL-c and TC levels in the AEG1, AEG2, and AEG3 groups were increased, and the TG in the AEG3 and AEG4 groups was increased remarkably (*p* < 0.05). There was no significant difference in the AEG1 and AEG2 groups (*p* > 0.05). Compared with the CON group, TG/HDL-c and TC/HDL-c of the AEG1 and AEG2 groups were significantly decreased (*p* < 0.05); TG/HDL-c of the AEG4 group was significantly increased, while TG/HDL-c of the AEG1 and AEG2 groups were significantly lower than the CON group. The TC/HDL-c in AEG1, AEG2, and AEG3 groups were significantly decreased (*p* < 0.05). In addition, LDL-c, NEFA (Non-esterified fatty acid), and MDH (Malic dehydrogenase) were not significantly different among all groups (*p* > 0.05).

### 3.3. Fat Tissue Histology

As shown in Table 7, in the subcutaneous back fat, the adipocyte area size and length-diameter ratio of the test groups were significantly lower than the CON group, while the number of adipocytes was increased remarkably (*p* < 0.05). There was no significant difference in length diameter and short diameter among all groups (*p* > 0.05). 

**Figure 1:**
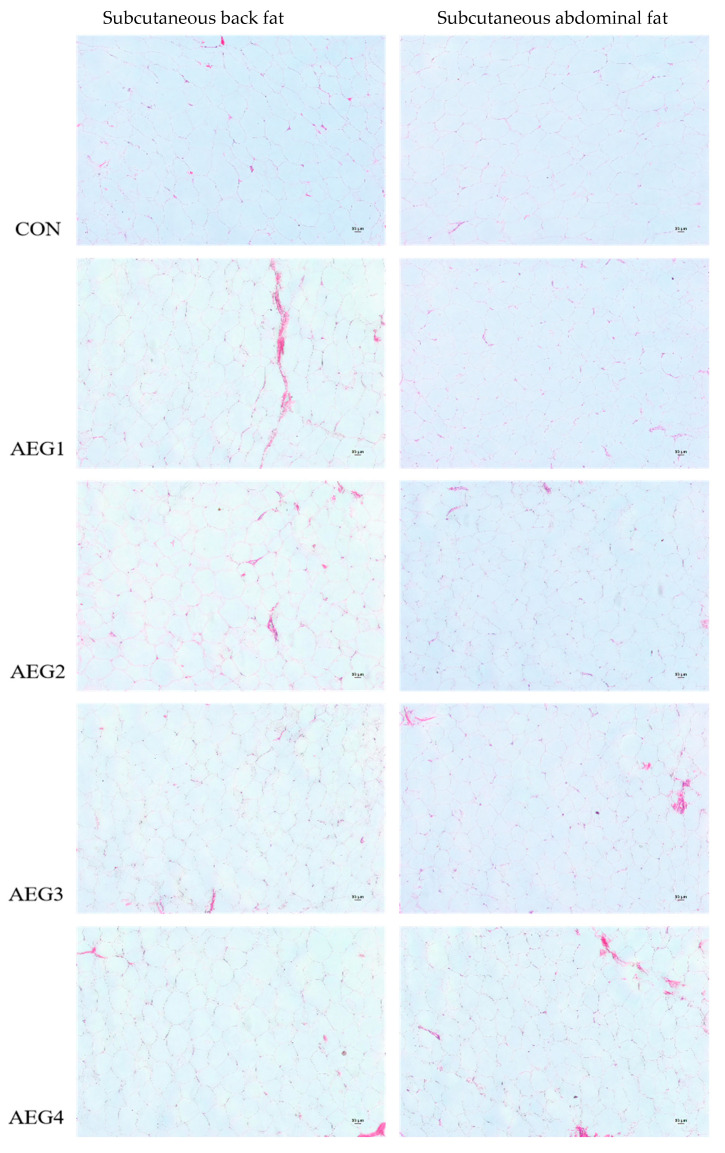
Subcutaneous adipose tissue morphology HE (Hematoxylin-Eosin staining) × 20.

In Figure 1 and Table 8, compared with CON group, the number of adipocytes in the subcutaneous abdominal fat of all test group pigs were significantly increased, but the area size and length diameter of cells were decreased remarkably; the length–diameter ratio of AEG1 and AEG2 group was significantly decreased (*p* < 0.05). There was no significant difference in the short diameter among all groups (*p* > 0.05).

### 3.4. FAs Composition

#### 3.4.1. FA Composition in Subcutaneous Back Fat

As shown in Table 9, there were no significant differences in SFAs, MUFAs, and PUFAs among all groups (*p* > 0.05).

Compared with the CON group, C10:0 in test groups were significantly decreased (*p* < 0.05). C12:0 and C14:0 in the AEG2 and AEG4 groups were observably lower than the CON, AEG1, and AEG3 groups. C15:0 in the AEG3 and C20:0 in the AEG1 groups were observably higher than all other test groups (*p* < 0.05). C16:0, C18:0, C22:0, and C23:0 was not significantly different among all groups (*p* > 0.05).

C14:1, C15:1, and C16:1 in the AEG4 group were significantly lower than those in other experimental groups, while C17:1 was significantly increased (*p* < 0.05). C20:1 was observably higher in the AEG1 group than in all other groups (*p* < 0.05). No significant differences were observed in C18:1 and C22:1 among the various groups. (*p* > 0.05).

C18:3n-6 was markedly elevated in the AEG2 group in comparison to all other groups (*p* < 0.05). C20:2n-6 in the AEG1 and AEG2 groups, C20:3n-6 in the AEG1 and AEG3 groups and C22:2n-6 in the AEG3 and AEG4 groups were significantly greater (*p* < 0.05). In addition, C20:3n-3 in the AEG4 group was observably lower (*p* < 0.05). C18:2n-6, C18:3n-3, C20:4n-6, C20:5n-3 and C22:6n-3 was not significantly different among all groups (*p* > 0.05).

#### 3.4.2. Composition of FAs in Subcutaneous Abdominal Fat

It can be seen from Table 10 that SFAs and MUFAs were not significantly different among all groups (*p* > 0.05). The PUFAs content in the AEG3 group was observably lower than all other groups *(p* < 0.05).

C10:0, C14:0, and C16:0 in the experimental groups were markedly lower than the CON group (*p* < 0.05). In addition, C17:0 in the AEG1 group was observably lower than all other groups, while C20:0 in the AEG1 group was significantly increased (*p* < 0.05). No significant differences were observed in C12:0, C15:0, C18:0, C22:0, and C23:0 among the various groups (*p* > 0.05).

C17:1 in the AEG4 group was observably increased than the CON, AEG1, and AEG2 groups (*p* < 0.05). In addition, C20:1 in the AEG1 group was significantly higher than the other experimental groups (*p* < 0.05). C15:1, C16:1, C18:1, and C22:1 was not significantly different among all groups (*p* > 0.05).

C18:2n-6 and C20:2n-6 in the AEG3 group and C18:3n-3 in the AEG1 group were prominently decreased than the other experimental groups, while C18:3n-6 in the AEG2 group was significantly higher than the other experimental groups (*p* < 0.05). C20:3n-3 in CON and AEG1 groups were observably increased than the other experimental groups (*p* < 0.05). No significant differences were observed in C20:3n-6, C20:4n-6, C20:5n-3, C22:2n-6, and C22:6n-3 among the various groups (*p* > 0.05).

### 3.5. Nutritional Indicators

#### 3.5.1. Subcutaneous Back Fat

As illustrated in Table 11, in the subcutaneous back fat, the LA/ALA (Linoleic acid/Alpha linolenic acid) value of the AEG1 group was notably greater than all other groups (*p* < 0.05). There were no significant differences in n-6: n-3, EPA + DHA (Eicosapentaenoic Acid+ Docosahexaenoic Acid), PUFAs: SFAs, IA, IT, UI, PI, HPI, NVI, and h/H among all groups (*p* > 0.05).

#### 3.5.2. Subcutaneous Abdominal Fat

Table 12 shows that the n-6: n-3 and LA/ALA values in subcutaneous abdominal fat were significantly higher in the AEG1 group compared to the CON group (*p* < 0.05). The PUFAs, SFAs, and PI values in the AEG3 group were significantly lower than the CON group (*p* < 0.05). Additionally, the NVI value was notably greater in the experimental groups than the CON group (*p* < 0.05). No significant differences were found in the values of EPA + DHA, IA, IT, UI, HPI, and h/H among all groups (*p* > 0.05).

### 3.6. Lipid Metabolism Gene Expression

#### 3.6.1. In Subcutaneous Back Fat

**Figure 2:**
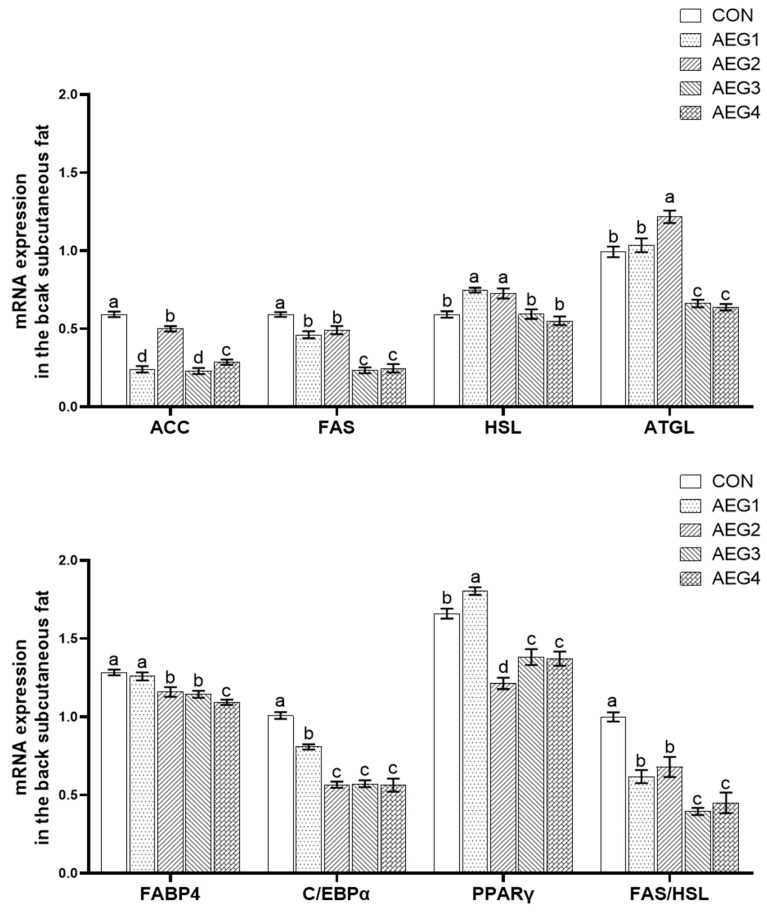
Lipid metabolism genes expression in subcutaneous back fat. The vertical bar represents the standard errors. The presence of lower-case letters indicate a statistically significant difference (*p* < 0.05).

As can be seen in Figure 2, the levels of ACC, FAS, C/EBPα genes, and FAS/HSL in experimental groups were significantly lower than the CON group (*p* < 0.05). Compared with the CON group, the expression levels of HSL in the AEG1 and AEG2 groups were significantly increased (*p* < 0.05), while the difference was not significant in the AEG3 and AEG4 groups (*p* > 0.05). In addition, the expression level of ATGL was higher in the AEG2 group and lower in the AEG3 and AEG4 groups (*p* < 0.05). The expression levels of ATGL and FABP4 genes in the AEG1 group were not significantly different from those in the CON group (*p* > 0.05). The expression levels of the PPARγ and FABP4 genes in the AEG2, AEG3, and AEG4 groups were significantly lower than those in the CON group (*p* < 0.05). The expression level of the PPARγ gene in the AEG1 group was significantly higher than the CON group (*p* < 0.05).

#### 3.6.2. In Subcutaneous Abdominal Fat

**Figure 3:**
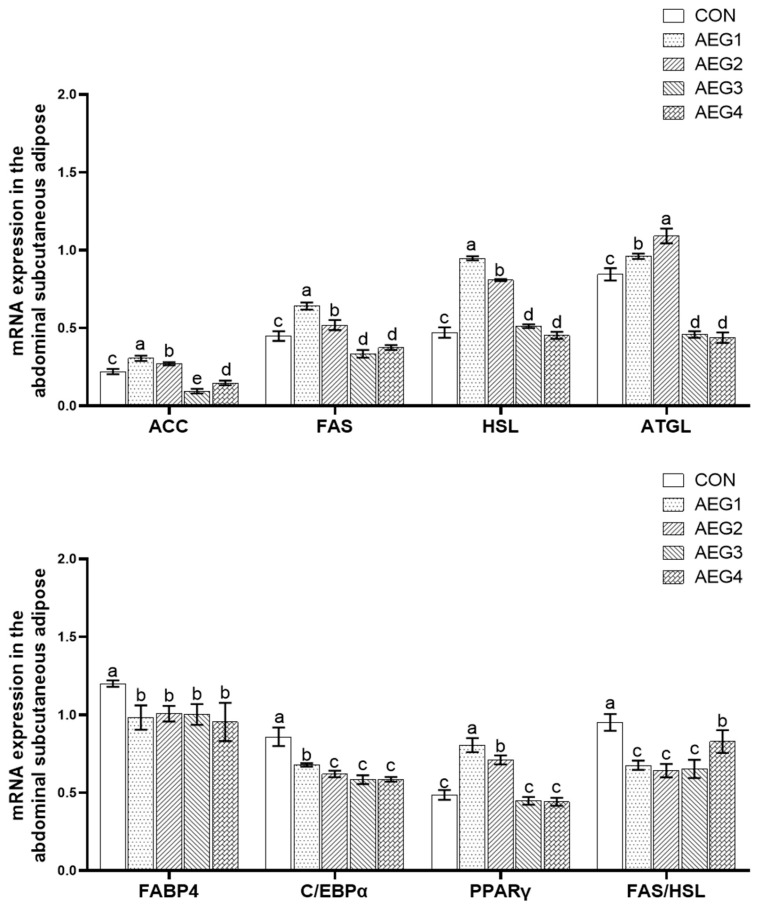
Lipid metabolism genes expression in subcutaneous abdominal fat. The vertical bar represents the standard errors. The presence of lower-case letters indicate a statistically significant difference (*p* < 0.05).

The result is shown in Figure 3 and, compared with the CON group, the expression levels of ACC, FAS, ATGL, PPARγ, and ATGL genes in the AEG1 and AEG2 groups were significantly increased, and the expression levels of ACC, FAS, and ATGL genes in AEG3 and AEG4 groups were significantly decreased (*p* < 0.05), while the expression levels of PPARγ and ATGL genes in AEG3 and AEG4 groups were not significantly different (*p* > 0.05). Furthermore, the levels of FABP4, C/EBPα, and FAS/HSL in the test groups were significantly lower than the CON group (*p* < 0.05).

## 4. Discussion

Slaughtering performance is an important indicator of meat production performance of livestock and poultry. This study found that there were no significant differences in backfat thickness, loin eye area, carcass weight, and slaughter rate among experimental groups compared with the CON group. Similar to our study, Tejeda’s study found no significant difference in carcass weight and slaughter rate between Iberian pigs fed acorns and grasses and standard diets [9]. Rey’s study found no significant difference in carcass weight between black Iberian pigs fed acorns and grasses [17]. In addition, we found that the lean meat rate in AEG2, AEG3, and AEG4 groups was notably greater than the CON group, while the lean meat rate of the AEG3 and AEG4 groups was lower than that of the AEG2 group, suggesting that acorn diets may inhibit fat deposition, and may improve muscle development to some extent. The reason may be that excessive acorns rich in tannic acid inhibit the absorption of nutrients from the intestine, thereby reducing the carcass quality and production performance of pigs [18,19,20]. However, the potential mechanism needs to be investigated by further studies.

From an adipose morphology perspective, fat deposition is primarily characterized by an increase in the number of fat cells during the developmental early stages and an increase in the volume of fat cells in the later stages of pig fattening [21]. Additionally, there are variations in the location of fat deposition in different parts of the body, such as perirenal and mesenteric fat depositing at a faster rate than subcutaneous fat [22]. The larger the area size and diameter of adipose tissue and a reduction in cell numbers per unit of the visual field, the greater ability to deposit fat. Pan’s study found that the area and diameter of obese Luchuan pigs were higher than that of lean Duroc pigs [23]. Previous research has shown that adding conjugated linoleic acid to the diet can help to reduce the area size of fat cells in subcutaneous adipose tissue in pigs, thereby inhibiting fat deposition [24]. This study discovered that the cell area size of subcutaneous back fat and subcutaneous abdominal fat was significantly lower than the CON group after feeding different proportions of acorn diets. Additionally, the number of cells increased in the unit field of view of microscope, indicating a reduction in the adipose deposition capacity in subcutaneous back fat and subcutaneous abdominal fat.

Fat deposition is affected by multiple factors. The content of triglycerides and total cholesterol in serum are important indicators of lipid metabolism [25]. The increase of TG and TC content will lead to the disorder of lipid metabolism in pigs, and then reduce the nutritional and economic value of pork [26]. The increase of HDL-C value can promote transport cholesterol metabolism, thus playing an important role in reducing obesity [27]. In the present study, the results indicate that acorns observably increased the serum TC and HDL-C content in the AEG1, AEG2, and AEG3 groups. Blood lipid levels are regulated by many factors and are in a dynamic process of change. Although the total cholesterol content has increased, the high-density lipoprotein content has also increased. TC/HDL value can be a more limited predictor of an important marker of coronary heart disease [28]. In addition, the TG/HDL value is an important marker for detecting the presence of insulin resistance [29,30]. Therefore, reducing TC/HDL and TG/HDL levels is of great significance to improve the blood lipid levels and health in pigs. Our results indicated that acorns could improve the health of pigs by regulating the serum TC/HDL and TG/HDL values of Yuxi black pigs.

The composition of FAs reflects the intake of dietary fatty acids and the endogenous processing of fats. The nutritional value of pork is affected by the difference in fatty acid composition. Nutritional indexes based on fatty acid composition can be used to evaluate the nutritional value of pork. NVI represents the potential effects of different lipid types on health, and the value of NVI is positively correlated with the quality of FAs [16]. Previous studies have shown that dietary acorns can reduce the SFAs content of fattening pigs [10]. The present study found that compared with the CON group, there were no significant differences in SFAs, MUFAs, and PUFAs of subcutaneous back fat and SFAs and MUFAs of subcutaneous abdominal fat in different groups. Inconsistent research results in the concentration of FAs might be due to the difference in the location of adipose tissue. We speculate that this may be caused by differences in the mechanisms involved in fatty acid synthesis and metabolism in different tissues. At present, there are few studies on the effect of acorns on fatty acid composition in subcutaneous fat, and the specific mechanism needs further study. We found that the NVI value of the abdominal subcutaneous adipose tissue in AEG2, AEG3, and AEG4 groups was significantly higher than that of the CON group, which suggested that feeding acorns may improve the nutritional value of the subcutaneous fat of Yuxi black pigs by increasing the NVI value.

The mechanism of lipid metabolism is very intricate, and the synthesis and decomposition of lipids are dynamic processes, which are influenced by the joint action of enzymes and genes. ACC and FAS are key rate-limiting enzymes involved in fatty acid synthesis. ACC is the first step of the fatty acid catalytic synthesis, and FAS is the last step of the fatty acid catalytic synthesis, both of which play an important regulatory role in fatty acid synthesis. Zhang’s research indicates that an increase in feed energy level leads to an increase in the expression levels of ACC and FAS genes that promote adipogenesis in adipose tissue, resulting in the promotion of fat deposition in the animal’s body. Conversely, a decrease in the expression levels inhibits fat deposition [31]. Zhao’s study found that curcumin is an effective inhibitor of FAS. Inhibiting FAS gene expression can prevent adipocyte differentiation and lipid accumulation [32].

Hormone-sensitive lipase (HSL) and fat triglyceride lipase (ATGL) are crucial enzymes involved in the breakdown of fats. HSL is an essential enzyme in lipid metabolism that hydrolyses monoacylglycerol, diacylglycerol, and triacylglycerol in adipose tissue to free fatty acids. It is a rate-limiting enzyme that plays a crucial role in the regulation of lipid metabolism [33]. Studies have shown that a reduction or absence of HSL expression can lead to an increase in triglyceride levels [34]. ATGL is a triglyceride hydrolase that selectively catalyzes the initial step of triglyceride hydrolysis, resulting in the production of DAG (triglyceride) and NEFA [35]. Adipose cells with increased ATGL expression exhibit enhanced lipolysis ability and reduced production of cellular lipid droplets [36].

FABP4 is a subtype of the fatty acid-binding protein family that primarily affects the transport, uptake, metabolism, and esterification of fatty acids. An increase in the expression level of FABP4 leads to an increase in fat deposition in the corresponding tissues [37]. PPARγ regulates adipogenesis, controlling the proliferation, differentiation, and fat deposition of adipocytes [38]. A reduction in PPARγ expression leads to decreased fat deposition, thereby regulating fat metabolism [39]. C/EBPα belongs to the CCAAT/enhancer binding protein family, which regulates adipogenesis by controlling glucose uptake and the expression of lipogenic genes in adipocytes. There are researchers who find that the expression of genes related to lipid droplet accumulation and lipid production is enhanced by the increase of C/EBPα expression, while the decrease of C/EBPα expression reduces the promoting effect of these genes [40].

Acorns are a rich source of polyphenols. As feed additives, polyphenols can regulate the expression of genes related to fat synthesis, decomposition, and transport, thereby affecting fat deposition in pigs. This study found that an acorn diet may down-regulate the expressions of ACC, FAS, FABP4, PPARγ, and C/EBPα genes in the AEG2 group, up-regulate the expressions of HSL and ATGL genes, and thus reduce the deposition of subcutaneous back fat in AEG2 group. At the same time, it can also be seen from FAS/HSL that the subcutaneous back fat of AEG1, AEG3, and AEG4 groups was inhibited. In subcutaneous abdominal fat, the expression levels of FABP4 and C/EBPα in test groups were markedly decreased compared with the CON group, and the expression levels of ACC, FAS, and PPARγ, involved in fat synthesis and HSL and ATGL involved in fat decomposition were up-regulated in AEG1 and AEG2 groups, indicating that the ability of fat decomposition and synthesis was improved. Moreover, in the AEG3 and AEG4 groups, the expression levels of ACC, FAS, PPARγ, HSL, and ATGL genes were significantly decreased, indicating that the fat synthesis and fat decomposition ability was decreased. However, fat synthesis and decomposition are dynamic processes, so the calculation of the ratio of FAS/HSL can better reflect the ability of fat deposition. Our study found that the FAS/HSL of subcutaneous abdominal fat in each experimental group was significant in comparison to the CON group, showing that the deposition of subcutaneous abdominal fat was reduced after feeding the acorn diet. Hence, acorn diets can adjust relating lipid metabolism genes and inhibit subcutaneous fat deposition.

## 5. Conclusions

In conclusion, a 30% acorn diet had positive effects on pork quality and the health of Yuxi black pigs by increasing the lean meat rate and the value of NVI and decreasing the values of TC/HDL and TG/HDL. Meanwhile, acorn diet could down-regulate fat synthesis genes and up-regulate lipolysis genes to inhibit subcutaneous fat deposition.

## Figures and Tables

**Table 1:** Acorn nutrient levels.

Nutrient Levels	Acorn
Water content (%)	18.37
Crude protein (%)	4.41
Crude fat (g/kg)	2.29
Crude ash (%)	2.09
Crude fibre (g/kg)	15.67
C18:0 (%)	1.91
C18:1 (%)	48.26
C18:2n-6 (%)	26.62
C18:3n-3 (%)	2.76
UFAs (%)	77.64
SFAs (%)	22.35

UFAs, unsaturated fatty acids; SFAs, saturated fatty acids.

**Table 2:** Diet composition and nutritional levels.

Items	Content				
	CON	AEG1	AEG2	AEG3	AEG4
Ingredient (%)					
Corns	65.51	51.32	44.92	38.70	32.14
Soybean meals	3.00	5.72	5.93	9.84	13.93
Wheat brans	22.24	14.28	7.52	3.00	0.00
Walnut dregs	5.32	4.72	7.73	4.48	0.00
Acorns	0.00	20.00	30.00	40.00	50.00
Premix	4.00	4.00	4.00	4.00	4.00
Total	100	100	100	100	100
Nutritional levels					
Metabolizable energy, MJ/kg	11.8	11.4	11.4	11.2	11.0
Crude protein (%)	11.9	11.4	11.49	11.38	11.08
Lysine (%)	0.5	0.5	0.5	0.5	0.6
Cysteine (%)	0.3	0.2	0.2	0.2	0.2
Methionine + Cysteine (%)	0.5	0.4	0.4	0.4	0.4
Protein energy ratio, g/MJ	10.1	10.0	10.1	10.2	10.1

Premix ingredients per kg: VA: 120,000.00 IU; VD3: 45,000.00 IU; VE: 700.00 IU; VK3: 45.00 mg; VB2: 150.00 mg; VB6: 50.00 mg; niacin amide: 750.00 mg; calcium pantothenate: 460.00 mg; choline chloride: 3.50 mg; copper: 0.31 g; iron: 3.50 g; zinc: 1.42 g; manganese: 0.83 g; iodine: 42.00 mg; selenium: 7.82 mg; ca: 16.00%; phosphorus: 3.53%; lys: 0.2%; cys: 0.05%; met: 0.1%.

**Table 3:** Dietary FAs content in each group.

Percentage of FAs (%)	Content				
	CON	AEG1	AEG2	AEG3	AEG4
C14:0	0.1	0.27	0.35	0.43	0.52
C16:0	12.27	13.08	13.08	13.63	14.36
C18:0	1.64	1.80	1.90	2.03	2.15
C18:1	23.53	24.96	25.73	26.70	27.61
C18:2n-6	56.88	55.64	55.32	54.42	53.41
C18:3n-3	2.83	2.90	3.04	2.93	2.79
UFAs	83.35	83.25	83.49	83.22	82.84
SFAs	14.28	13.91	13.55	13.56	13.63
U/S	5.84	5.98	6.16	6.14	6.08
n-6	56.88	55.64	55.32	54.42	53.41
n-3	2.83	2.90	3.04	2.93	2.79
n-6/n-3	20.10	19.19	18.20	18.57	19.14

UFAs, unsaturated fatty acids; SFAs, saturated fatty acids; U/S, unsaturated fatty acids/saturated fatty acids.

**Table 4:** Primer sequences for RT-qPCR.

Genes	GenBank	Primer Sequence (5′ to 3′)	Proudunt Length, bp
ACC	AF175308.1	F: CCTCTGCCTTCTGACATGCTGAC	305
		R: GCCAGTCCGATTCTTGCTCCAC	
ATGL	EU373817.1	F: GGGTCTGCCTGGGTGATACTGG	374
		R: GGTGATGGTGCTCTTGAGTTCGTAG	
FABP4	NM_001002817.1	F: AAGAAGTGGGAGTGGGCTTTGC	320
		R: AATTCTGGTAGCCGTGACACCTTTC	
FAS	NM_213839.1	F: CATCGTGAGGGTCAATTCTGCTGTC	338
		R: CATTTGGTGTTGCTGGTTGGTGTG	
HSL	AF141958.1	F: CTTTGCGGGTATTCGGGAACAGG	212
		R: TGTGGCTTGTGCGGAAGAAGATG	
PPARγ	NM_214379.1	F: GCAGGAGCAGAGCAAAGAGGTG	345
		R: GCCAGGTCGCTGTCATCTAATTCC	
C/EBPα	XM_003127015.4	F: CCCGCACTTGCAGTTCCAGATC	245
		R: ACTCGTTGCTGTTCTTGTCTACCG	
GAPDH	NM_001206359.1	F: CAAGGCTGTGGGCAAGGTCATC	279
		R: AAGTGGTCGTTGAGGGCAATGC	

**Table 5:** Effect of acorns on slaughter performance.

Items	CON	AEG1	AEG2	AEG3	AEG4	SEM	*p*
Mean backfat thickness (cm)	3.315	3.186	3.052	3.566	3.238	0.354	0.434
Loin eye area (cm^2^)	70.605	72.458	74.176	71.742	71.419	6.169	0.096
Carcass weight (kg)	105.133	104.700	107.333	104.233	103.567	1.608	0.253
Dressing percentage (%)	70.737	67.503	76.564	75.631	75.319	5.791	0.500
Lean meat rate (%)	42.113 ^c^	43.001 ^c^	48.370 ^a^	46.287 ^b^	46.267 ^b^	0.522	0.001

In the same row, differences in lower case letters indicate a statistically significant difference (*p* < 0.05).

**Table 6:** Effects of feeding acorn diet on blood lipid indexes of Yuxi black pigs.

Items	CON	AEG1	AEG2	AEG3	AEG4	SEM	*p*-Values
Low-density lipoprotein-cholesterol (mmol/L)	1.009	1.341	1.594	1.375	1.341	0.175	0.081
High-density lipoprotein-cholesterol (mmol/L)	0.405 ^c^	0.795 ^b^	1.118 ^ab^	1.235 ^a^	0.409 ^c^	0.153	0.001
Triglyceride (mmol/L)	0.342 ^b^	0.385 ^b^	0.519 ^ab^	0.763 ^a^	0.721 ^a^	0.117	0.015
Total cholesterol (mmol/L)	2.145 ^c^	2.826 ^ab^	3.240 ^a^	3.125 ^ab^	2.474 ^bc^	0.283	0.016
Non-esterified fatty acid (mmol/L)	0.578	0.569	0.504	0.442	0.418	0.119	0.583
Malic dehydrogenase (U/mgprot)	0.377	0.393	0.395	0.341	0.305	0.031	0.067
TG/HDL-c	0.889 ^b^	0.488 ^c^	0.495 ^c^	0.617 ^bc^	1.742 ^a^	0.165	0.001
TC/HDL-c	5.412 ^a^	3.619 ^b^	2.982 ^b^	2.538 ^b^	6.401 ^a^	0.782	0.002

In the same row, differences in lower case letters indicate a statistically significant difference (*p* < 0.05). TG/HDL-c, Triglyceride divided by high-density lipoprotein-cholesterol; total cholesterol divided by high-density lipoprotein-cholesterol.

**Table 7:** Effect on subcutaneous back fat tissue morphology after feeding acorn diets.

Items	CON	AEG1	AEG2	AEG3	AEG4	SEM	*p*-Values
Adipocytes number (10^2^)	1.60 ^b^	1.78 ^a^	1.87 ^a^	1.80 ^a^	1.84 ^a^	0.05	0.004
Adipocytes area (μm^2^)	4556.94 ^a^	3748.62 ^b^	3387.62 ^c^	3381.91 ^c^	3785.17 ^b^	124.14	0.001
length diameter (μm)	98.28	86.77	93.80	92.91	90.26	4.03	0.136
Short diameter (μm)	62.24	60.86	66.72	64.12	63.20	2.49	0.26
Long/short diameter ratio	1.58 ^a^	1.43 ^b^	1.41 ^b^	1.45 ^b^	1.43 ^b^	0.026	0.001

In the same row, differences in lower case letters indicate a statistically significant difference (*p* < 0.05).

**Table 8:** Effect on subcutaneous abdominal fat tissue morphology after feeding acorn diets.

Items	CON	AEG1	AEG2	AEG3	AEG4	SEM	*p*-Values
Adipocytes number (10^2^)	1.71 ^c^	2.22 ^a^	2.26 ^a^	2.25 ^a^	2.04 ^b^	0.06	0.01
Adipocytes area (μm^2^)	4496.54 ^a^	3110.48 ^b^	3211.95 ^b^	3258.86 ^b^	3107.43 ^b^	201.74	0.001
length diameter (μm)	96.50 ^a^	79.45 ^b^	85.30 ^b^	83.91 ^b^	84.56 ^b^	4.45	0.033
Short diameter (μm)	61.42	58.68	62.39	57.38	55.37	3.05	0.21
Long/short diameter ratio	1.57 ^a^	1.35 ^b^	1.37 ^b^	1.55 ^a^	1.53 ^a^	0.029	0.001

In the same row, differences in lower case letters indicate a statistically significant difference (*p* < 0.05).

**Table 9:** The impact of acorns on the composition of FAs in subcutaneous back fat tissue.

Items	AEG Level (%)					SEM	*p*-Value
	CON	AEG1	AEG2	AEG3	AEG4		
SFAs							
C10:0	0.065 ^a^	0.053 ^b^	0.055 ^b^	0.056 ^b^	0.047 ^c^	0.002	0.001
C12:0	0.090 ^a^	0.084 ^a^	0.060 ^b^	0.085 ^a^	0.043 ^c^	0.004	0.001
C14:0	1.544 ^a^	1.517 ^a^	1.321 ^b^	1.530 ^a^	1.159 ^c^	0.049	0.001
C15:0	0.066 ^bc^	0.056 ^c^	0.076 ^b^	0.082 ^a^	0.076 ^ab^	0.005	0.004
C16:0	23.816	22.028	21.824	22.895	24.624	2.143	0.663
C17:0	0.278 ^c^	0.231 ^c^	0.354 ^b^	0.411 ^ab^	0.451 ^a^	0.030	0.001
C18:0	11.882	10.190	14.406	13.850	11.964	2.463	0.478
C20:0	0.152 ^b^	0.231 ^a^	0.070 ^c^	0.072 ^c^	0.091 ^c^	0.017	0.001
C22:0	0.112	0.117	0.104	0.129	0.100	0.015	0.409
C23:0	0.259	0.261	0.265	0.264	0.212	0.044	0.711
MUFAs							
C14:1	0.041 ^a^	0.042 ^a^	0.040 ^a^	0.041 ^a^	0.035 ^b^	0.001	0.002
C15:1	0.034 ^a^	0.041 ^a^	0.030 ^ab^	0.044 ^a^	0.017 ^b^	0.006	0.010
C16:1	4.625 ^a^	4.488 ^ab^	4.090 ^b^	4.423 ^ab^	3.496 ^c^	0.185	0.001
C17:1	0.326 ^d^	0.294 ^d^	0.440 ^c^	0.506 ^b^	0.564 ^a^	0.024	0.001
C18:1	40.410	41.954	39.529	39.401	41.196	2.887	0.881
C20:1	0.889 ^b^	1.209 ^a^	0.920 ^b^	0.871 ^b^	0.894 ^b^	0.058	0.001
C22:1	0.102	0.109	0.101	0.120	0.111	0.012	0.487
PUFAs							
C18:2n-6	13.114	14.731	13.695	12.707	12.586	0.875	0.169
C18:3n-6	0.543 ^c^	0.575 ^c^	0.780 ^a^	0.687 ^b^	0.559 ^c^	0.038	0.001
C18:3n-3	0.265	0.171	0.258	0.258	0.232	0.042	0.233
C20:2n-6	0.576 ^bc^	0.718 ^a^	0.658 ^ab^	0.562 ^c^	0.626 ^bc^	0.037	0.011
C20:3n-6	0.062 ^b^	0.083 ^a^	0.052 ^b^	0.077 ^a^	0.062 ^b^	0.006	0.003
C20:3n-3	0.188 ^a^	0.205 ^a^	0.210 ^a^	0.189 ^a^	0.154 ^b^	0.013	0.012
C20:4n-6	0.106	0.119	0.132	0.138	0.119	0.017	0.448
C20:5n-3	0.084	0.086	0.093	0.078	0.088	0.021	0.963
C22:2n-6	0.244 ^c^	0.250 ^bc^	0.315 ^ab^	0.334 ^a^	0.361 ^a^	0.031	0.011
C22:6n-3	0.127	0.157	0.127	0.188	0.133	0.022	0.082
SFAs	38.265	34.768	38.529	39.375	38.766	3.115	0.619
MUFAs	46.428	48.137	45.151	45.407	46.313	2.975	0.864
PUFAs	15.307	17.095	16.320	15.218	14.921	1.009	0.244

The amount of each fatty acid was calculated as peak area percentage of total fatty acids. In the same row, differences in lower case letters indicate a statistically significant difference (*p* < 0.05).

**Table 10:** Effect of acorns on FA composition in subcutaneous abdominal fat.

Items	AEG Level (%)					SEM	*p*-Value
	CON	AEG1	AEG2	AEG3	AEG4		
SFAs							
C10:0	0.075 ^a^	0.062 ^cd^	0.067 ^b^	0.059 ^d^	0.064 ^bc^	0.001	0.001
C12:0	0.092	0.088	0.068	0.069	0.058	0.014	0.133
C14:0	1.749 ^a^	1.610 ^b^	1.447 ^d^	1.518 ^c^	1.247 ^e^	0.012	0.001
C15:0	0.080	0.072	0.068	0.063	0.078	0.009	0.383
C16:0	25.305 ^a^	24.230 ^b^	22.847 ^d^	23.841 ^c^	23.035 ^d^	0.153	0.001
C17:0	0.350 ^ab^	0.217 ^c^	0.337 ^b^	0.312 ^b^	0.412 ^a^	0.031	0.001
C18:0	12.757	14.299	14.758	19.664	16.445	3.040	0.274
C20:0	0.113 ^b^	0.214 ^a^	0.069 ^c^	0.073 ^c^	0.084 ^c^	0.011	0.001
C22:0	0.107	0.123	0.108	0.102	0.106	0.014	0.639
C23:0	0.298	0.362	0.261	0.310	0.313	0.040	0.227
MUFAs							
C14:1	0.046 ^ab^	0.047 ^ab^	0.043 ^bc^	0.053 ^a^	0.037 ^c^	0.004	0.021
C15:1	0.037	0.032	0.030	0.031	0.023	0.006	0.234
C16:1	5.091	5.297	4.391	5.450	4.802	0.325	0.055
C17:1	0.374 ^c^	0.264 ^d^	0.400 ^bc^	0.456 ^ab^	0.478 ^a^	0.031	0.001
C18:1	38.480	38.148	39.171	35.436	38.109	2.991	0.768
C20:1	0.778 ^c^	1.089 ^a^	0.781 ^c^	0.970 ^b^	0.804 ^c^	0.048	0.001
C22:1	0.117	0.109	0.124	0.094	0.084	0.021	0.318
PUFAs							
C18:2n-6	11.932 ^a^	11.673 ^a^	12.602 ^a^	9.357 ^b^	11.690 ^a^	0.841	0.028
C18:3n-6	0.449 ^bc^	0.423 ^c^	0.646 ^a^	0.442 ^bc^	0.515 ^b^	0.036	0.001
C18:3n-3	0.270 ^a^	0.134 ^c^	0.291 ^a^	0.208 ^b^	0.218 ^b^	0.010	0.001
C20:2n-6	0.498 ^a^	0.511 ^a^	0.501 ^a^	0.448 ^b^	0.510 ^a^	0.020	0.047
C20:3n-6	0.073	0.075	0.068	0.079	0.063	0.007	0.245
C20:3n-3	0.263 ^a^	0.250 ^a^	0.226 ^b^	0.223 ^b^	0.224 ^b^	0.009	0.003
C20:4n-6	0.093	0.105	0.117	0.110	0.098	0.015	0.561
C20:5n-3	0.093	0.091	0.086	0.103	0.084	0.012	0.547
C22:2n-6	0.310	0.299	0.326	0.346	0.247	0.037	0.161
C22:6n-3	0.168	0.171	0.167	0.184	0.162	0.014	0.604
SFAs	40.927	41.277	40.030	46.013	41.852	3.022	0.374
MUFAs	44.924	44.993	44.940	42.489	44.338	2.975	0.904
PUFAs	14.149 ^a^	13.730 ^a^	15.030 ^a^	11.499 ^b^	13.811 ^a^	0.868	0.024

The amount of each fatty acid was calculated as peak area percentage of total fatty acids. In the same row, differences in lower case letters indicate a statistically significant difference (*p* < 0.05).

**Table 11:** Effect of acorns on dietary fat quality indicators of subcutaneous back fat.

Items	AEG Level (%)					SEM	*p*-Value
	CON	AEG1	AEG2	AEG3	AEG4		
n-6: n-3	16.065	19.970	17.302	14.901	17.624	3.868	0.748
LA/ALA	54.284 ^b^	86.870 ^a^	53.899 ^b^	49.337 ^b^	54.193 ^b^	8.163	0.006
PUFAs: SFAs	0.401	0.492	0.424	0.392	0.392	0.045	0.211
EPA + DHA (%)	0.211	0.244	0.220	0.266	0.221	0.022	0.170
IA	0.486	0.431	0.441	0.482	0.486	0.054	0.741
IT	1.144	0.987	1.157	1.211	1.189	0.162	0.675
UI	79.069	84.486	80.142	78.314	78.197	3.608	0.433
PI	19.149	21.221	20.498	19.682	18.817	1.133	0.271
HPI	2.058	2.322	2.268	2.089	2.159	0.212	0.690
NVI	2.195	2.367	2.471	2.326	2.257	0.228	0.786
h/H	2.197	2.508	2.413	2.236	2.269	0.250	0.703

In the same row, differences in lower case letters indicate a statistically significant difference (*p* < 0.05).

**Table 12:** Effect of acorns on dietary fat quality indicators of subcutaneous abdominal fat.

Items	AEG Level (%)					SEM	*p*-Value
	CON	AEG1	AEG2	AEG3	AEG4		
n-6: n-3	16.818 ^bc^	20.292 ^a^	18.554 ^ab^	15.034 ^c^	19.100 ^ab^	1.321	0.020
LA/ALA	44.218 ^b^	88.149 ^a^	43.439 ^b^	44.839 ^b^	53.615 ^b^	5.678	0.001
PUFAs: SFAs	0.345 ^a^	0.333 ^a^	0.377 ^a^	0.251 ^b^	0.337 ^a^	0.033	0.031
EPA + DHA (%)	0.261	0.262	0.253	0.286	0.246	0.018	0.281
IA	0.547	0.522	0.478	0.555	0.487	0.027	0.057
IT	1.262	1.294	1.224	1.565	1.353	0.171	0.357
UI	75.415	74.501	77.389	67.700	74.074	3.267	0.110
PI	18.249 ^a^	17.702 ^a^	19.332 ^a^	15.641 ^b^	17.785 ^a^	0.908	0.027
HPI	1.829	1.915	2.095	1.805	2.075	0.107	0.061
NVI	2.025 ^c^	2.165 ^b^	2.360 ^a^	2.312 ^a^	2.369 ^a^	0.028	0.001
h/H	1.945	2.008	2.232	1.851	2.138	0.125	0.075

In the same row, differences in lower case letters indicate a statistically significant difference (*p* < 0.05).

## Data Availability

Raw data supporting the conclusions of this paper will be provided by the corresponding author.
